# New Insights into the Control of Cell Fate Choices and Differentiation by Retinoic Acid in Cranial, Axial and Caudal Structures

**DOI:** 10.3390/biom9120860

**Published:** 2019-12-11

**Authors:** Heidrun Draut, Thomas Liebenstein, Gerrit Begemann

**Affiliations:** Developmental Biology, University of Bayreuth, Universitätsstraße 30, 95447 Bayreuth, Germany; Heidrun1.Draut@uni-bayreuth.de (H.D.); thomas.liebenstein@uni-bayreuth.de (T.L.)

**Keywords:** retinoic acid, spinal cord, neuromesodermal precursors, notochord, vertebrae, calvaria, osteoblasts, teeth, dentition, regeneration

## Abstract

Retinoic acid (RA) signaling is an important regulator of chordate development. RA binds to nuclear RA receptors that control the transcriptional activity of target genes. Controlled local degradation of RA by enzymes of the Cyp26a gene family contributes to the establishment of transient RA signaling gradients that control patterning, cell fate decisions and differentiation. Several steps in the lineage leading to the induction and differentiation of neuromesodermal progenitors and bone-producing osteogenic cells are controlled by RA. Changes to RA signaling activity have effects on the formation of the bones of the skull, the vertebrae and the development of teeth and regeneration of fin rays in fish. This review focuses on recent advances in these areas, with predominant emphasis on zebrafish, and highlights previously unknown roles for RA signaling in developmental processes.

## 1. Introduction

All-*trans*-retinoic acid (RA) is a small molecule that is critical during developmental processes of chordate embryos. It is of great importance that RA is available in exactly the right places and at appropriate concentrations; therefore, a precise regulation of RA signaling is indispensable for development. RA is thought to control the activities of more than 500 genes [1,2]. RA is a lipophilic molecule derived from retinol (vitamin A) and is relatively short lived. It can be inactivated locally (see below), and can either act directly on the cell that produces it (cell-autonomous and autocrine) or on cells neighboring the source of synthesis (non-cell-autonomous and paracrine). Together, these properties make RA well suited to act as a diffusible morphogen in several developmental processes [3]. Disruption of RA signaling during critical developmental stages results in a wide range of defects, for example, in the facial region, eyes, inner ear, heart, lungs, forelimbs and many other organs [4,5,6].

Dietary sources of vitamin A mainly consist of retinol and retinyl ester or are ingested in the form of carotenoids, which have to be converted to vitamin A in the intestine and other tissues [7]. Following uptake by intestinal cells, a fraction of the provitamin A carotenoids is cleaved into retinal by the cytoplasmic protein β-carotene-15, 15′-monooxygenase (BCMO1, also known as BCO1). BCMO1 is a key component of a regulatory network that controls the absorption of carotenoids and fat-soluble vitamins [8]. Retinal can then be converted to retinol, which is intracellularly sequestered by cellular retinol-binding protein type I (CRBPI) and esterified into retinyl esters for storage, mainly by lecithin retinol acyltransferase (LRAT) [9,10,11]. Although most retinoids can diffuse through cell membranes without any carrier protein, vitamin A is mobilized from the liver by retinol binding protein (RBP) and delivered to other organs via the circulation. In the plasma, RBP4 binds to vitamin A and this complex is bound by transthyretin (TTR), which enhances binding in the complex. Interestingly, in fish, RBP carries vitamin A without forming a complex with TTR [12]. At target cells, vitamin A is released from RBP by its receptor, stimulated by retinoic acid 6 (STRA6), a transmembrane protein that is believed to transport vitamin A through a pore into the cytoplasm [13,14,15].

Retinol is oxidized sequentially in two steps, first to all-*trans*-retinaldehyde, catalyzed primarily by the retinol dehydrogenase RDH10. The reverse reaction, from all-*trans*-retinaldehyde back to retinol, is carried out by DHRS3 [16,17,18,19]. The second step is catalyzed by retinaldehyde dehydrogenases (ALDH1A1, -A2 and -A3), produces RA and is not reversible. In early embryonic development, the main RA producing enzyme is ALDH1A2, while the other isoforms contribute to more elaborately regulated patterns of RA synthesis during organ development [20]. RA signaling activity is mainly controlled at the levels of RA synthesis and degradation. Bioactive RA is metabolized through 4-hydroxilation into various polar compounds by either of 3 isoforms of CYP26 proteins, called CYP26A1, -B1 and -C1, whose expression is regulated in a cell-type-specific manner [4]. CYP26 activity counteracts the biological activity of RA [21,22], yet some of the emerging metabolites (4-oxo-RA, 4-OH-RA and 5,6-epoxy-RA) display RA-similar activities: they are able to rescue vitamin-A-deficient quails when administered exogenously and to modulate Cyp26 gene expression, suggesting that in vivo they may be further oxidized to inactive forms [23].

Thus, RA availability is controlled by the regulated expression of RDH10 and ALDH1A1-A3 enzymes for RA synthesis and DHRS3 and the CYP26s for reduction of all-*trans*-retinaldehyde and the depletion of bioactive RA, respectively [24]. In further layers of complexity, appropriate levels of RA signaling are provided by feedback mechanisms that couple reductions in RA signaling to transcriptional upregulation of RDH10 and the ALDH1A isoforms [25,26]. Feedback regulation can lead to overcompensation scenarios where the application of teratogenic levels of RA results both in the expected gain-of-function phenotypes and loss-of-function effects due to excessive upregulation of CYP26A1 [27]. Intracellularly, cellular RA-binding proteins (CRABP-I and -II) associate with RA and translocate it to the nucleus or shunt available RA to CYP26s. CRABPs are able to compensate for changes in RA synthesis and contribute to signaling robustness [28].

RA is the major and endogenous agonist for the different RA receptors (RARs), all of which are members of the nuclear receptor superfamily [29], and are called RARα, RARβ and RARγ in mammals. RARs heterodimerize with retinoid X receptors (RXRs) and bind DNA at retinoic acid response elements (RAREs). Generally, in ray-finned fish, the orthologous RAR or RXR genes are one of numerous examples of genes that exist in multiple copies, created by genome duplications during evolution of these fish species. Each gene copy is characterized by a distinct expression pattern indicating an individual function [30,31,32]. RAR/RXR heterodimers are widely expressed in various tissues (typical examples being the head mesenchyme, the forebrain and the tail) and knockout/knockdown studies have found evidence of widespread functional redundancies between the different heterodimers [30,33,34]. All-*trans*-retinoic acid exhibits very little binding to RXR [35], but another RA isoform, 9-*cis*-retinoic acid, can act as an RXR-specific ligand in vitro. However, it remains controversial whether 9-*cis*-retinoic acid is a universal ligand of RXRs in vivo. In addition, endogenous 9-*cis*-retinoic acid is below detection levels in most mammalian tissues with the exception of the mouse pancreas [36]. Lastly, 13-*cis*-retinoic acid is a naturally occurring form of retinoic acid that is found in blood and tissues of vertebrates, but it has no described endogenous regulatory function [37].

In the canonical model of RA signaling, RAR/RXR dimers bind to RAREs in the absence of RA and recruit transcriptional corepressor complexes, which themselves attract chromatin modifiers that keep the promoter in a repressed (heterochromatin) state, so that transcription is not possible [38]. In the presence of RA, the molecule binds to RAR, which triggers conformational changes that result in corepressor release and binding of coactivators instead [5]. Coactivators recruit diverse complexes of proteins that alter the chromatin structure of the target gene promoter region to an active state. Activated RARs will then recruit the transcription machinery to the target gene promoter. RA also recruits further RAR/RXR dimers to previously unbound RAREs by as yet unknown mechanisms. Transcription ends when activated RARs attract coregulators that again recruit chromatin-modifying proteins that end RA activity or when RARs are degraded by proteasomes [39]. However, it should be noted that a small but growing number of examples has been identified in which binding of RA leads to silencing of gene activation in developmental processes [40,41,42]. RA has also been shown to mediate non-genomic effects that do not affect gene expression directly, by rapidly and transiently activating several kinase cascades. For example, several cell types activate the p38 mitogen-activated protein kinase (p38MAPK) in response to RA. Here, RARs have been found to be present outside the nucleus and are often associated with the plasma membrane [39,43].

Retinoid signaling has been shown to play important roles in cellular differentiation processes [44,45,46,47]. Many recent review articles are available that highlight specific roles of RA in animal development: Considerable progress was made in understanding how RA acts in shaping developing organs through the identification and subsequent functional characterization of the genes involved in RA metabolism, retinoid transport, cellular uptake and delivery to the nucleus [19,41,48,49]. Concise overviews have been published with intermittent updates on the various roles of RA in embryonic and male germ cell development [5,24,50,51], others outline the importance of RA signaling gradients for patterning processes in the embryo and their elaboration by metabolic processes of RA synthesis and catabolism [5,21]. Excellent reviews are available with a focus on the development of individual organs, such as the heart and head [21,52,53], hematopoiesis [54], the nervous system [55] and the maintenance of post-natal bone [56]. Lastly, particular attention has been given in recent years to the roles of RA signaling in modulating the immune response [57,58,59,60,61].

Here, we review developmental processes in which either considerable progress has been made recently towards a better understanding of the roles that RA plays or where a body of work has accumulated that warrants a synopsis to put the new findings into perspective. We provide examples from different areas of developmental biology, embryonic development and regeneration, on how gradients of RA signaling are established and maintained to control cell fate decisions. The first focus is on developing neuromesodermal precursors of the vertebrate embryo, where a rostral to caudal RA signaling gradient is established during somitogenesis that acts on the rostral presomitic mesoderm and the neural tube. It is antagonised by a Wnt/Fgf signaling gradient emanating from more caudal structures and sets up a signaling front that determines whether presomitic mesodermal cells become competent to respond to signals from the segmentation clock, a molecular oscillator, and initiate somite formation. The same RA gradient controls cell fate decisions in the adjacent neural tube. The recurrent theme of RA acting through a gradient is taken up again towards the end, when we examine how proliferating osteogenic cells in the regenerating fin can undergo controlled redifferentiation to bone-forming osteoblasts. While the somite patterning process also informs the segmented pattern of the vertebrae that gives rise to the skeleton of the spinal column in mammals, zebrafish embryos show that vertebrae formation can also be dependent on the notochord [62]. However, vertebral bodies (centra) are formed by two different mechanisms in amniotes and anamniotes. In mammals and birds, the vertebral column derives from endochondral ossification. Sclerotome-derived mesenchymal cells migrate around the notochord and differentiate either into chondrocytes, which establish a segmented cartilage scaffold, or into osteoblasts, which mineralize the cartilage scaffold to eventually form the centra [63,64]. In contrast, teleost vertebral body precursors develop through intramembranous ossification via mineralization of the notochord sheath [63,64,65,66,67].

As an entry point into a more detailed look at the roles played by RA in skeletal development, we summarize the evidence that the initial steps of vertebrae formation require signaling from RA and its local degradation by Cyp26b1. Two other processes that require RA and that shape the zebrafish head skeleton and hard tissues are the formation of the calvaria, i.e., the bones of the upper skull, and the development of the pharyngeal teeth. We summarize new findings from zebrafish with relevance to human diseases that examine the phenotypes caused by altered RA signaling on the lineage leading from mesenchymal stem cells to bone-forming (osteogenic) cells during calvarial development. The formation of the first teeth in zebrafish embryos is meaningful from an evolutionary perspective, because its dependency on RA appears to be an acquired trait that is not present outside the cyprinid family, to which zebrafish belong. Finally, some of the roles for RA in embryonic osteogenic cells are reprised in osteoblasts of larvae and adults. However, osteoblasts are capable of dedifferentiation to preosteoblasts, contribute to proliferating cells in the regenerating fin and then redifferentiate in the appropriate spatial patterns to rebuild the injured fin. We summarize various essential roles that RA plays to orchestrate the events required to lead osteoblasts through the regeneration process. We end our review with an update on the hypothesis that RA provides positional memory in the zebrafish caudal fin. Further evidence has accumulated now that correlates RA with position-dependent proliferation rates rather than the position-defining activity itself.

## 2. RA Signaling Controls Induction and Differentiation of Neuromesodermal Progenitors

**Figure 1:**
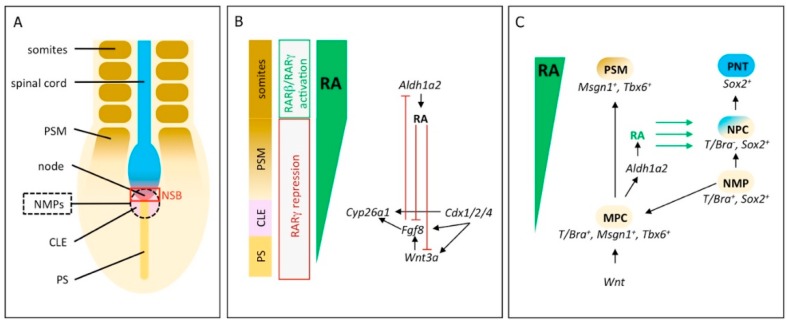
RA signaling controls induction of NMPs, their differentiation into neural lineage and somitogenesis. (**A**) Schematic representation of the caudal region of an E7.5–E9.5 gastrulating mouse embryo to visualize the location of neuromesodermal progenitors (NMPs). NMPs are located in the caudal lateral epiblast (CLE) and the node-streak border (NSB). (**B**) Interactions between RA, FGF and Wnt signaling during body axis elongation and somitogenesis. RA produced in the CLE, pre-somitic mesoderm (PSM) and the somites and *Cyp26a1* counteracting from the distal notochord and chordoneural hinge (CNH; at E9.5–E14.5; not shown in Figure 1A) establish a gradient of RA. A feedback mechanism between RA and *FGF/Wnt* signaling plays a key function in axis elongation and somitogenesis. *Cdx* genes additionally act on Wnt, FGF and RA signaling to adjust the levels of RA. Studies in *Xenopus* showed that during axis elongation, RARs act as transcriptional activators and repressors, dependent on the amount of RA present in the system. (**C**) The role of RA in NMP induction and differentiation. Upon migration, NMPs (*T/Bra*^+^/*Sox2*^+^) differentiate to neural or mesodermal progenitor cells (NPC and MPC). MPC (*T/Bra*^+^/*Msgn1*^+^/*Tbx6*^+^) express *Aldh1a2*, leading to enhanced RA production, which diffuses to the surrounding tissue and results in repression of *T/Bra* and activation of *Sox2* in NPC and, therefore, to neural differentiation. Figure modified from [68,70,71,72,73,74]. Additional abbreviations: PS, primitive streak; PNT, pre-neural tube.

Neuromesodermal progenitors (NMPs) play a central role during body axis elongation in vertebrates. They are a transient population of bipotential cells located in the caudal lateral epiblast (CLE), the node-streak border (NSB) and the chordoneural hinge (CNH) and are able to differentiate into mesodermal or neural tissue (Figure 1A) [68]. Generally, there are two populations of NMPs called expanding- and depleting-NMPs. Expanding-NMPs are a self-renewing cell population that is only found in amniote embryos and that is responsible for the formation of the spinal cord in the trunk region. In contrast, depleting-NMPs form the tail spinal cord and are completely depleted at the end of somitogenesis. In anamniote embryos, the blastopore closes after gastrulation, followed by the formation of the tailbud. Due to these differences, anamniotes do not require expanding-NMPs and control body elongation through depleting-NMPs [68,69].

NMPs are characterized by the co-expression of the transcription factors *T/Bra* and *Sox2*. Their differentiation process into either neural or mesodermal cells is a complex process of regulatory mechanisms, where the fate of cells highly depends on their position in the progenitor region [68,75,76,77,78]. In mouse embryos, the gene encoding the RA synthesizing enzyme *Aldh1a2* is transiently expressed in the posterior mesendoderm as well as in primitive streak and node cells at E7.5 and E7.75 and later, in the pre-somitic mesoderm (PSM) and in mature somites [79,80]. A feedback mechanism between RA and FGF signaling is a key regulator in body axis extension and somitogenesis. In this context, RA plays a permissive role by repressing caudal *Fgf8* and *Wnt8* expression [38,81,82,83]. In chick and mouse embryos (HH10 or E8.5–E9.5, respectively), *Fgf8* negatively influences RA signaling by inhibition of *Aldh1a2* expression and activation of *Cyp26a1* expression, to ensure that the caudal-most region of the CLE and the NSB are free of RA or receive only low RA concentration (Figure 1B) [78,84,85,86].

The role of RA in NMP establishment and differentiation, however, only recently became evident. Most studies that address this question are based on embryonic stem cells (ESC) that were differentiated to NMPs in vitro [70,87,88]. To elucidate endogenous RA target genes during NMP differentiation, mouse NMPs were exposed in vitro to a 2 h treatment with an RA concentration that mimics physiological conditions (25 nM). This setup avoided the identification of false targets that occurs at unphysiologically high (1 µM) RA concentrations and through the analysis of cell types, such as ESCs, that normally are not exposed to RA in vivo. Whole-transcriptome analysis showed that this immediately activates numerous RA-responsive genes—*Cdx1*, *Sox2*, *Nkx1.2*, *Fgf15*, *Zfp503* and *Gbx2* among others—indicating an instructive role of RA. At the same time, the treatment resulted in the repression of a large number of other targets, for example, *Wnt8a*, *Fgf8*, *Id1* and *Fst*. *Id1* encodes a transcription factor activated by bone morphogenetic protein (BMP) signaling and *Fst* encodes the BMP antagonist Follistatin. Expression studies concerning these two genes on wildtype and *Aldh1a2^−/−^* mouse embryos revealed that RA limits expression of *Id1* to mesoderm progenitors at the caudal tip of the embryo by suppressing *Id1* in the NMP niche. Similarly, RA is required to eventually extinguish *Fst* in the CLE and presomitic mesoderm when somitogenesis commences. Therefore, RA separates both genes’ activity from the NMP area, indicating a permissive role for RA in NMP differentiation during mouse embryonic development [70]. The simultaneous activation of genes associated with neural (*Nkx1.2*, *Fgf15*) and mesodermal lineages (*Zfp503*, *Gbx2*) suggests that RA acts on the posterior neuroectoderm as well as on presomitic mesoderm formation [70].

In addition to its effects on NMP differentiation, another role of RA in NMP induction was recently discovered [71]. Removal of all RA signaling in vitro, by cultivation of *Aldh1a2^−/−^* mouse ESC in the absence of vitamin A, disturbed the formation of *T/Bra^+^*/*Sox2^+^* NMP cells. The treatment led to a downregulation of *Sox2* expression, but induced *T/Bra*, *Msgn1*, *Tbx6* as well as *Eomes* and *Mixl1*, indicating a mesodermal character [71,89]. On the other hand, the addition of high levels of RA (10 or 100 nM) to differentiating NMPs in vitro blocked mesoderm induction and promoted neural differentiation towards pre-neural tube (PNT) identity, as evidenced by the expression of *Sox2*, *Sox1* and *Nkx1.2* [71,87,90,91]. These results suggest that mesodermal identity (*T/Bra^+^*/*Tbx6^+^*/*Cdx^−^*) is established in the absence of RA signaling, the induction of NMP identity (*T/Bra^+^*/*Sox2^+^*) is mediated by low levels of RA and high levels of RA induce pre-neural identity (*Sox2^+^*/*Nkx1.2^+^*) [71,77]. Considering the consequences of these findings for the in vivo system, it is assumed that a rostral-to-caudal gradient of RA signaling influences the induction and positioning of distinct trunk progenitors [71]. The gradient is established by RA produced in the CLE, the PSM and the somites and by *Cyp26a1* counteracting from the distal notochord and CNH (Figure 1B) [77,80]. To generate a feedback mechanism that regulates the outcome of NMP differentiation, mesoderm markers *Msgn1* and *Tbx6*, themselves activated by Wnt signaling and *T/Bra*, mediate the upregulation of *Aldh1a2* to increase RA synthesis, leading to the repression of *T/Bra* and the activation of *Sox2* and finally, to neural differentiation (Figure 1C) [92,93,94].

Another component of RA signaling regulation are the genes of the *Cdx* family. These encode homeobox transcription factors and play a developmental role in axis elongation. Their primary region of expression is in the primitive streak and later, in development in the tailbud of the embryo [95,96]. To study their role in NMPs, mouse stem cells lacking all three paralogous *Cdx* genes (*Cdx^1,2,4−/−^*) were created and cultivated in NMP-inducing conditions [71]. This resulted in strong induction of *Aldh1a2* expression, significant downregulation of *Cyp26a1* expression and the loss of *Wnt3a* and *Fgf8* expression—circumstances that would promote neural tissue formation. On the other hand, the inhibition of RA signaling in *Cdx^1,2,4−/−^* cells by treatment with the pan-RAR inverse agonist BMS493 resulted in mesodermal cell formation. However, neither treatment led to the differentiation of NMP cells, suggesting that *Cdx* genes are required to act on Wnt, FGF and RA signaling to achieve the correct RA levels that are needed to promote the induction of NMPs and their subsequent differentiation (Figure 1B). In contrast to mice, RA is not required for extension of the body axis in zebrafish, an organism that is lacking expanding-NMPs. It seems that the repressive effect of RA on caudal *Fgf8* is only acting in expanding-NMPs and, therefore, restricted to higher vertebrates [71]. These differences demonstrate that caution is advised when transferring knowledge achieved from one model organism to another.

RARs play important activating or repressing roles—strictly depending on local levels of RA, —in the differentiation process of NMPs to unsegmented PSM and finally, to mature somites. RARβ and RARγ are expressed in the caudal tail and trunk area in mammals [5]. In *Xenopus* embryos, the predominant isoform expressed throughout the entire caudal region of the embryo, including PSM and CNH, is *rarγ2*. This receptor acts as both an activator and a repressor [72]. In the transition region, where PSM cells are differentiating towards somitic mesoderm, the presence of RA is indicated by *aldh1a2* expression. Here, Rarγ2 acts as an activator to promote somitomere differentiation [72,97]. However, in the areas of unsegmented PSM and CNH cells, RA is absent or present at low concentrations owing to *cyp26a1* expression. This allows Rarγ2 to act as a repressor to maintain the pool of mesodermal progenitor cells (Figure 1B). A potential target that is repressed by Rarγ2 might be *ripply2*, a repressor of *tbx6*; therefore, promoting *tbx6* expression [98,99]. Similar results were obtained in a study that differentiated mouse ESCs via Wnt pathway activation [100]. Here the pan-RAR inverse agonist AGN193109, which stabilizes the heterodimeric complex of RA receptors (RAR/RXR) with their transcriptional co-repressors, was applied to inhibit RA signaling during ESC differentiation, beginning at a differentiation stage that corresponds to cells from the CLE. This promoted the formation of the paraxial mesoderm, characterized by the upregulation of the gene markers *Tbx6* and *Msgn*. A continued treatment with the inverse agonist eventually repressed the maturation of PSM into the somitic mesoderm. This suggests that RARs function in epiblast and early mesoderm progenitor cells—areas where RA is absent—to promote their differentiation into paraxial mesoderm lineage [100].

In contrast to that, *rarβ2* expression is sensitive and responsive to RA. This is the receptor subtype most strongly downregulated by pan-RAR inverse agonist AGN193109 and correspondingly upregulated in response to a treatment with the pan-RAR agonist TTNPB. Initiation or maintenance of *rarβ2* expression is dependent on Rarα/γ, as a knockdown of either of those two receptors causes the loss of *rarβ2* expression [73]. In *Xenopus*, this RA receptor is active in mature somites and its loss leads to the rostral expansion of unsegmented PSM markers (*tbx6*, *msgn*, *fgf8*) and also shifts the expression domains of somitomere markers (*ripply2*, *mespa*) rostrally. As a result, fewer but larger somites develop that lack distinct boundaries and chevron morphology. Therefore, in *Xenopus*, RA activates Rarβ2 in the trunk to regulate somitogenesis, while *rarγ2* is expressed in the RA free tail area, sustaining the PSM and NMP cell population [72,73] (Figure 1B).

## 3. Initiation of Vertebrae Formation in Zebrafish Relies on Precisely Regulated RA-Signaling

The early development of the vertebral column has been shown to be dependent on precise RA-signaling in both mammals and fish [61,68,69,70,71]. The vertebral column is a segmented axial supporting structure that consists of alternating vertebral bodies (centra) and intervertebral discs. In tetrapods, the skeletal elements of the vertebral bodies develop from sclerotome-derived cells by endochondral ossification [72,73,74]. In contrast, vertebral bodies of teleosts develop through intramembranous ossification in two steps. First, vertebral body precursors (chordacentra) form through segmented mineralization of the notochord sheath by cells of the underlying notochord, called chordoblasts [75,76,77,78,79,80]. Subsequently, sclerotome-derived cells are recruited around the notochord sheath, which differentiate into two different types of osteoblasts: One class of osteoblasts is located in the middle and on the anterior and posterior edges of the chordacentra and is responsible for the secretion and mineralization of extracellular bone matrix to form the surrounding centra. A second type of osteoblasts has been identified in medaka that is situated within the intervertebral regions and is involved in the deposition of the collagenous matrix of the extra elastica, thus preventing mineralization [63,101,102]. Excess RA could lead to a transition of the collagenous matrix depositing osteoblasts to matrix mineralizing osteoblasts/osteocytes. However, this hypothesis remains to be tested.

Several studies suggest that the segmentation process and formation of these chordacentra is dependent on spatially and temporally distinct gene expression and patterning mechanisms that are not determined by the intrinsic segmentation clock [62,102,103,104]. In the earliest stages of zebrafish vertebrae development, chordoblasts are uniformly distributed over the collagenous notochord sheath [102,105]. At the onset of chordacentra formation, the expression of chordoblast markers (like *col2a1a* and *col9a2*) is downregulated in an alternating, ring-shaped pattern, beginning anteriorly and sequentially moving posteriorly along the axis [102,104,105,106]. Concomitantly, expression of *entpd5a*, a marker for biomineralizing activity in zebrafish [107], is upregulated in the same cells. Ultimately, osteoblasts are recruited to the mineralized sheath domains to form the vertebral bodies [102,104].

In zebrafish, the onset of *entpd5a*-expression and mineralization of the chordoblasts is dependent on Notch-signaling as well as precisely regulated RA-signaling [102,104]. While an excess of RA leads to an expanded, stronger and often even fused expression of *entpd5a* along the anteroposterior notochord axis, inhibition of RA-synthesis using the Aldh-Inhibitor DEAB abolishes *entpd5a* expression and prevents the reiterative axial mineralization [104]. In Japanese flounder, *Paralichthys olivaceus*, treatment with an excess of RA similarly induces the narrowing and fusion of centra, combined with a complete loss of notochord and intervertebral tissues within fused centra [108]. The repetitive, RA-sensitive areas in zebrafish are precisely defined through a negative feedback mechanism, in which RA is thought to activate expression of both *cyp26b1* and *entpd5a*. The immediate activation of *cyp26b1* might drive a fast degradation of RA (the exact source of which is not currently known), thus impeding RA to spread into adjacent, prospective intervertebral regions. This, in turn, might be a crucial mechanism for the establishment of alternating zones of mineralizing and non-mineralizing, cartilage-like domains. Accordingly, expression of *cyp26b1* along the notochord is strongly and rapidly upregulated after addition of excess RA and eliminated upon DEAB-treatment, indicating regulation by RA [104].

Mineralizing chordoblasts in zebrafish larvae reduce collagen 2 production over time, as indicated by reduced *col2a1a*-expression. This downregulation is mimicked by treatment with RA at earlier stages of development also and extends along the entire anteroposterior notochord axis, including the prospective intervertebral areas, and results in overall mineralization [104,106]. In contrast, complete, as well as chordoblast-specific, inhibition of RA-signaling results in an evenly distributed *col2a1a* expression and at the same time, loss of *cyp26b1*-signaling and mineralization [104]. Considering the reduced matrix production in combination with the morphological changes from roundish-compact to more stellate-like-shaped chordoblasts, a reduction of endoplasmic reticulum and an, overall, slightly thinner notochord sheath [104], the impact of RA on chordoblasts is reminiscent of the effects of RA on osteoblasts and preosteocytes during intramembranous bone formation (see below) [109,110,111,112]. Taken together, RA is involved in orchestrating the repeated pattern along the anteroposterior notochord axis and simultaneously regulates the first steps towards centra development. Future studies should address the question if chordoblasts are also involved in centra formation in other vertebrates [104] and, therefore, if the molecular mechanisms of the two different ossification processes are conserved in amniotes and anamniotes. Even though centra in tetrapods are formed by endochondral ossification of sclerotome-derived cartilaginous templates on the outer surface of the notochordal sheath, a simultaneous contribution of chordoblasts from the inner side of the notochordal sheath has not been addressed to date [104].

## 4. RA Controls Cell Fate Determination during Calvarial Bone Development

**Figure 2:**
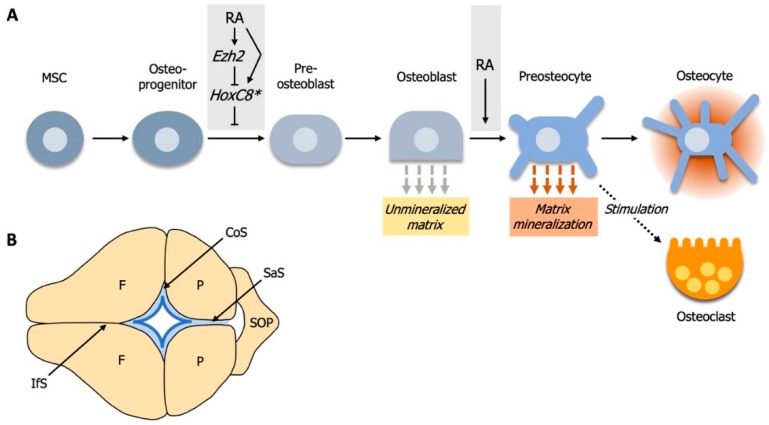
Differentiation process from mesenchymal stem cells (MSC) to mature osteocytes. (**A**) In mice, RA and *Ezh2* are required to act simultaneously, yet with opposing effects on anti-osteogenic factors (*) for early calvarial bone lineage commitment. At later differentiation stages, in mice and zebrafish, RA is required for the transition from osteoblasts to preosteocytes. Excess RA results in premature matrix mineralization and increased stimulation of osteoclasts. (**B**) Structure and development of the calvarial plates in mouse and zebrafish (anterior is to the left). Expression of *cyp26b1* (dark blue) at the osteogenic fronts (light blue) during calvarial growth indicates the necessity of downregulated RA-signaling for accurate calvarial development. Further abbreviations: CoS, coronal suture; F, frontal bone; IfS, interfrontal suture; P, parietal bone; SaS, sagittal suture; SOP, supraoccipital bone.

RA signaling plays important roles during the development of the vertebrate skull. This is exemplified by various calvarial malformations and diseases that are associated with RA-signaling disorders [111,113,114,115,116]. The cranium represents the upper part of the skull that encloses and protects the brain and is divided into the cranial base and the calvarium. The calvarial bones are joined through sutures and, as they are made up of flat bones, arise through intramembranous ossification. During this process, mesenchymal stem cells (MSC) differentiate into osteoblasts, and subsequently, to preosteocytes and osteocytes, which together compose an aggregation of osteogenic cells [109,110,117] (Figure 2A). MSCs can also enter a chondrocyte- or a odontoblast-fate, all of which are regulated by *Runx2* [118,119,120]. In addition, a complex network of interactions with components of several other signaling pathways, like FGF-, BMP-, Wnt- and thyroid hormone-signaling pathways is necessary for accurate linage commitment and cell differentiation during bone development [118,119,120,121,122,123,124,125,126,127,128]. While bone forming osteoblasts are of cuboidal shape and important for the secretion of non-mineralized bone matrix (osteoid), preosteocytes stimulate matrix mineralization and assume a shape that is more similar to osteocytes. Eventually, mature osteocytes are located in lacunae, embedded in the mineralized bone matrix with a stellate-like shape and long cell protrusions [109,110]. The maintenance and remodeling of bone requires the activity of osteoclasts—multinucleated cells of hematopoietic origin [129]—that are believed to be in crosstalk with osteoblasts and osteocytes [109,130,131,132].

### 4.1. Elevated RA-Signaling Leads to Premature Osteoblast to Preosteocyte Transition

Human patients carrying a null or hypomorphic mutation in the gene encoding the RA-degrading enzyme CYP26B1 exhibit seemingly contradictory craniofacial anomalies like calvarial bone hypoplasia (reduced formation and fragmentation of bone) and craniosynostosis (premature ossification of sutures), respectively. Recent findings have started to unravel the mechanisms behind these developmental defects [111,112,133]. Similar to the human hypomorphic patients, the hypomorphic *cyp26b1* zebrafish mutant *stocksteif* (*sst*) also displays premature fusion of calvarial sutures through premature suture matrix mineralization. Osteoblasts normally reside at the osteogenic fronts of growing calvaria and later, within the sutures. After suture formation, *cyp26b1* expression is faintly detectable at the edges of the calvarial plates, which is consistent with observations in newborn mice [111] (Figure 2B). However, in zebrafish *sst* mutants or after treatment with RA shortly before suture formation, premature synostosis (fusion) of the coronal suture initiates bilaterally at the edges of the frontal and parietal calvarial plates, which coincides with sites of *cyp26b1* expression in the wildtype condition [111]. Furthermore, expression levels of the osteoid collagen genes *col1a1* and *col10a* are reduced in sutural osteoblasts of zebrafish *sst* mutants and the morphology of these cells has shifted from an osteoblastic globular shape towards a more (pre-) osteocyte stellate-like shape [111]. Treatment of murine MC3T3 preosteoblasts with RA also leads to a dose-dependent reduction of osteoblast marker expression, while expression-levels of osteocyte markers are progressively upregulated. Since cell number, proliferative activity and apoptosis of sutural cells in *sst* mutant zebrafish are not significantly altered, these observations suggest that *cyp26b1* hypomorphic defects result from a loss of osteoblastic characteristics, especially the production of matrix osteoid at the edges of the calvarial plates, and a gain of (pre-) osteocyte characteristics of sutural cells, which is accompanied by premature mineralization. Accordingly, partial loss of *cyp26b1* activity causes coronal craniosynostosis through accelerated osteoblast to (pre) osteocyte transition [111].

Human CYP26B1 null patients and *Cyp26b1^−/−^* homozygous mice exhibit fragmentated calvarial bones, a seemingly opposite cranial defect to those occurring in zebrafish *cyp26b1* hypomorphic mutants [111,112,114]. Comparable calvarial fragmentation phenotypes have been induced in zebrafish larvae treated with exogenous RA during early calvarial plate development, which results in a reduction in bone formation at the calvarial osteogenic fronts and in the thickness of calvarial plates [112]. This is consistent with findings obtained in mice that were fed vitamin A, which resulted in less dense calvarial bones accompanied with overall reduced bone areas [116]. Observations of the calvarial plates of wildtype zebrafish larvae and mice revealed *cyp26b1* expression in central parts and on the outer surfaces of the calvarial plates, while the expression of *aldh1a2* is largely restricted to meninges cells underneath the calvarial plates and close to the active osteogenic fronts [112,134,135]. This indicates that RA-signaling is active in areas of calvarial growth, as bone formation proceeds preferentially at the inner calvarial surface during vertical growth, and reduced RA-signaling in areas where calvarial growth is diminished.

Increased levels of RA have no effects on the number of osteogenic cells in zebrafish, neither around the coronal suture of *cyp26b1/sst* mutants nor at the osteogenic fronts after RA-treatment [111,112]. However, osteogenic cells, particularly at the calvarial tips, change in shape to flat and elongated forms, while bone-lining cells downregulate the expression of osteoblast-markers in favor of preosteocyte markers [112]. Thus, similar to the coronal suture, RA-treatment triggers the premature osteoblast to preosteocyte transition at calvarial osteogenic fronts.

The likely cause of calvarial plate fragmentation in RA-treated zebrafish is an active loss of mineralized matrix. Calvarial fragmentations are associated with high activity of bone-resorbing osteoclasts [112] and likewise, in mice fed excess vitamin A, the number and activity of osteoclasts increases on the inner, endocranial surface of calvarial bones [116]. Excess vitamin A causes an enlargement of blood vessels and an increase of cells positive for Icam1, a key endothelial molecule involved in active recruitment of osteoclast precursors, in the thoroughly perfused dura mater membrane that lies beneath the osteoclast-rich endocranial bone surface. As osteoclast precursors originate from hematopoietic cells of the monocyte/macrophage line [136], vitamin A is likely to increase adhesion and transendothelial migration of osteoclast precursors [116].

During normal bone remodeling, osteogenic cells can regulate the activity of osteoclasts and vice versa through an increased production of stimulators or inhibitors [137]. These are secreted or cell surface-tethered cytokines or bone matrix components that serve as ligands to osteoblast- or osteoclast-bound receptors and result in enhanced bone formation or resorption [112,137]. Promotors and inhibitors from osteoblasts include M-CFS, MCP-1, RANKL, LPA and OPG, Ephrin B2, SEMA3A, respectively, while CC3, EPHB4, CTHRC1 and ATP6V0D2, SEMA4D, sclerostin, miR-214-3p represent known promotors and inhibitors from osteoclasts, respectively [137]. Observations in mice and zebrafish after vitamin A/RA-treatment revealed a strong physical association between preosteocytes and osteoclasts on the endocranial surface of the calvarial plates, supporting the notion that these two cell types interact with each other [112,116]. As in the coronal suture and the calvarial osteogenic fronts, premature osteoblast to preosteocyte transitioning is strongly prominent after RA-treatment at sites of calvarial fragmentation. Thus, more osteoclasts can be activated and recruited. This indicates that preosteocytes play an essential role during the RA-induced and osteoclast-dependent calvarial fragmentation [112].

In the osteoclast-deficient *pfeffer* mutant [138,139], treatment with RA fails to induce bone resorbing activity or the fragmentation of frontal plates, while the expression of genes encoding for osteoclast-stimulating ligands in preosteocytes is induced in the same way as in wildtype zebrafish [112]. However, RA-treatment of zebrafish after targeted ablation of *osx*-positive osteogenic cells [140], which includes osteoblasts, neither leads to calvarial fragmentations nor to an upregulation of genes encoding for osteoclast-stimulating ligands. Hence, RA acts on the osteogenic cell lineage to attract osteoclasts. The finding that osteogenic cells are the primary target of RA-signaling is further supported by the observation that the RA-target gene *cyp26b1* [141] is expressed in osteogenic cells rather than in osteoclasts [112]. In conclusion, RA-signaling influences osteoclasts not directly, but via osteogenic cells during calvarial bone resorption.

Taken together, the seemingly contradictory cranial developmental defects (craniosynostosis versus calvarial bone hypoplasia and fragmentation) observed after exposure to elevated RA-levels can be explained by the RA-induced dose- and stage-dependent differentiation of matrix-producing osteoblasts to mineralizing (pre-) osteocytes [111,112]. While the reduced calvarial size results from a decrease in osteoid production due to a premature differentiation from osteoblasts to mineralizing preosteocytes, the calvarial fragmentation is caused by an increased number of preosteocyte-stimulated osteoclasts. In *cyp26b1* hypomorphs, Cyp26b1 levels are still sufficient for the adequate horizontal growth of the frontal calvarial plates, while the elevated RA-level at the sutures leads to the appearance of prematurely differentiated (pre-) osteocytes and hence, to premature suture matrix mineralization and calvarial fusion [111,112]. This might explain why *cyp26b1* amorphs do not display craniosynostosis, as the frontal and parietal calvarial plates are reduced in size and, therefore, not able to form a proper suture.

### 4.2. RA-Signaling and Ezh2 Act in Opposition for Calvarial Bone Lineage Commitment

During early calvarial bone development, RA signaling and the histone methyltransferase *Ezh2* (*enhancer of zeste homolog 2*) are required to be active simultaneously but with opposing effects for early calvarial bone lineage commitment [142]. The Polycomb Repressive Complex 2 (PRC2) is a multi-protein complex and epigenetic regulator that requires RA for recruitment to specific genes [42,142,143]. EZH2, the catalytic component of PRC2, mediates the trimethylation of histone 3 on lysine 27 (H3K27me3), which leads to transcriptional repression of target genes and is required for neural-crest-derived cartilage and bone formation [142,144,145]. Mutations in the human EZH2 gene cause Weaver syndrome, which is characterized by overgrowth, advanced bone age and craniofacial defects, like domed heads and smaller mandibles [142,146,147,148,149]. Conditional mutation of *Ezh2* in mouse (further referred to as *Ezh2* mutant) cranial mesenchymal stem cells prior to skull bone cell fate selection in vivo revealed a stage-specific and transient role of *Ezh2* for proper skull bone development. *Ezh2* mutant mice displayed decreased craniofacial bone volume and size, but almost no effects on cell proliferation, cell survival and specification of early calvarial bone precursors [142]. Instead, *Ezh2* is required for the commitment to an osteoblast-fate, as the number of OSX-positive osteogenic cells is strongly reduced. These phenotypes are highly reminiscent of the effects caused by hypervitaminosis A or treatment with vitamin A or RA in humans, mice and zebrafish, respectively [111,112,114,116].

Further experiments showed that RA gavage leads to an upregulation, and RA-signaling inhibition to a reduction, of *Ezh2* expression [142]. In conclusion, both conditional *Ezh2*-mutation and elevated RA-signaling cause the reduction of OSX-positive osteogenic cells and thus calvarial bone development, while RA directly regulates *Ezh2* expression (Figure 2A). This mode of action can be described as an “incoherent type-1 feedforward model” (I1-FFL), where two arms act in opposition, while one positively regulates the other [150]. In this case, RA signaling positively and EZH2 negatively regulate the expression of anti-osteogenic factors to stimulate calvarial bone formation. RA signaling inhibition in *Ezh2* mutant mice leads to a partial rescue of the parietal and occipital bones as well as OSX expression, thus demonstrating that simultaneous inhibition of the positive and negative arm of the I1-FFL is able to partially rescue posterior calvarial bone formation [142]. In a candidate approach to identify anti-osteogenic factors regulated by EZH2 and RA, *HoxA1*, *HoxC8* and *Hand2* were found to exhibit the most notable increases in *Ezh2* mutant mice and were also significantly upregulated after RA exposure. Furthermore, concurrent application of RA to *Ezh2* mutant mice considerably increases the expression levels of these anti-osteogenic factors, whereas simultaneous RA-inhibition in *Ezh2* mutants reduces and thus re-establishes the number of anti-osteogenic factor HOXC8-positive cells in the parietal bone primordia [142]. Thus, stage-specific *Ezh2* expression and tight control of RA-signaling levels are required to synergistically regulate the expression of anti-osteogenic factors and hence to ensure accurate calvarial bone lineage commitment.

## 5. RA Controls the Development and Number of Pharyngeal Teeth in Zebrafish

A well-documented effect of RA in mammalian tooth development is to antagonize hard tissue mineralization, but there is no in vivo model to support a more basic role in tooth formation [151]. However, examining the roles of RA in zebrafish tooth formation illuminates how evolutionary modifications of RA-mediated gene regulation can facilitate diversity in vertebrate dentition. The family of cyprinids, of which the zebrafish is a member, only develop pharyngeal teeth and their main tooth row generally has five teeth. The majority of ray-finned fish (actinopterygians) develop either oral teeth, which are placed around the mouth opening, or pharyngeal teeth, which are situated on the fifth ceratobranchial bone in the back of the pharynx, or both. RA has been shown to fine-tune tooth number at a microevolutionary scale within this taxonomic group. This idea is supported by various observations: There is variation in tooth number in a few cyprinid species that exist with either four or six teeth [152,153] and RA-treatment in goldfish, a cyprinid with four teeth, produces an extra tooth [154]. Also, RA-treated zebrafish embryos will frequently develop a sixth tooth in the main row of teeth. This phenotype is also observed in heterozygous zebrafish of the *stocksteif (sst*) mutant, which harbor a mutation in *cyp26b1*, which causes a physiologically more subtle elevation of RA concentration [154]. The Mexican tetra (of the order Characiformes), a close relative of zebrafish (Cypriniformes), and medaka, a more distantly related species in the beloniform order, differ from zebrafish in possessing oral teeth in addition to pharyngeal teeth. Surprisingly, in these species, the formation of both types of dentition is independent of RA. It is likely that RA-induction of teeth in Cypriniformes is an evolutionary-derived trait that is correlated with a shift of *aldh1a2* expression as a precondition to regulation of pharyngeal tooth development. This newly gained dependency on RA may have played a role in the evolutionary loss of oral teeth in zebrafish and all other cyprinids [155].

How does RA control tooth number in cyprinid fish? The first pair of teeth (named 4V1), differentiates at 48 hours post fertilization (hpf). Its appearance is followed by the formation of a pair of neighbors, 3V1 medially and 5V1 laterally. 4V1 is replaced by 4V2 at 12 days post-fertilization and in adult fish, the fifth branchial arch has grown to accommodate eleven teeth in a stereotypical arrangement, with five teeth positioned in the ventral main row, four teeth in a medio-dorsal row and two teeth in the most dorsal row [156]. Tooth development through “first generation teeth” like 4V1 is representative for many other families of actinoptrygians. Where it occurs, the first tooth has been proposed to determine the formation of the remaining teeth of a row.

At the time of first tooth bud formation *aldh1a2* and the RA receptors *raraa* and *rarab* are expressed broadly in the ventral pharynx, but *aldh1a2* expression is excluded from the developing 4V1 tooth bud mesenchyme. Furthermore, tooth bud mesenchyme expresses *cyp26b1* to protect itself from RA [154]. An experimental increase in RA signaling activity expands the expression of markers of the dental (*dlx2*, *lhx6*) and pharyngeal mesenchyme (*pitx2a*). The consequences are a widened expression domain of tooth markers in the ventral fifth ceratobranchial arch that generates an expanded domain competent for tooth induction [154]. Induction of 4V1 is dependent on sequential signaling first by RA and then, FGFs between 43 and 49 hpf [155,157] and has been shown to determine the formation of the remaining teeth of a row: Application of antagonists of either RA- or FGF signaling, after 4V1 is induced, suppresses the development of the adjacent germs of 3V1 and 5V1 [158]. Timed early treatments with exogenous RA from 24 to 36 (or 52) hpf also induces ectopic 4V1 teeth in more anterior and dorsal positions of the pharynx, where teeth are normally absent [159]. Such ectopic 4V1 tooth germs initiate their own new rows of teeth, starting with neighboring 3V1 and 5V1 teeth. 4V1 expresses Fgfs (*fgf4* and/or *fgf3*) and Fgf receptors are expressed in pharyngeal arches of both wildtypes and RA-treated embryos with ectopic 4V1 tooth germs [157,158]. FGF signaling plays an activating role in tooth formation; therefore, Fgfs are good candidates for initiating dental rows in zebrafish [160]. The epistatic relationships between RA and FGF signaling are not fully resolved, as RA does not rescue early tooth markers in the absence of FGF signaling and overexpression of *fgf10* is ineffective in rescuing tooth development when RA is absent, even though *fgf10* is sufficient to induce some ectopic teeth [155].

Like RA, induced deficiency of thyroid hormones also generates supernumerary teeth anterior to the beginning of a tooth row [161], which may guide future explorations into the underlying mechanisms. Thyroid hormone receptors and RARs share RXRs as heterodimeric partners, which is thought to explain why RA and thyroids repress the activation of each other’s target genes in craniofacial neural crest cells (from which the tooth-producing odontoblasts derive) [162,163,164]. It is conceivable that a reduction of thyroid hormones allows a more ready activation of RA signaling [161]. Further evidence for cross-talk between RA and thyroid hormone signaling comes from mouse F9 cells, which serve as an in vitro model of embryonic stem cell differentiation. Here, RA promotes thyroid hormone uptake through the transcriptional up-regulation of a thyroid hormone transporter gene (*Mct8*) [165]. It thus remains to be tested if the regulation of tooth formation by RA involves modulation of thyroid hormone signaling.

## 6. Essential Roles for RA in Zebrafish Fin Regeneration

### 6.1. RA Controls Blastema Formation and Maintenance

**Figure 3:**
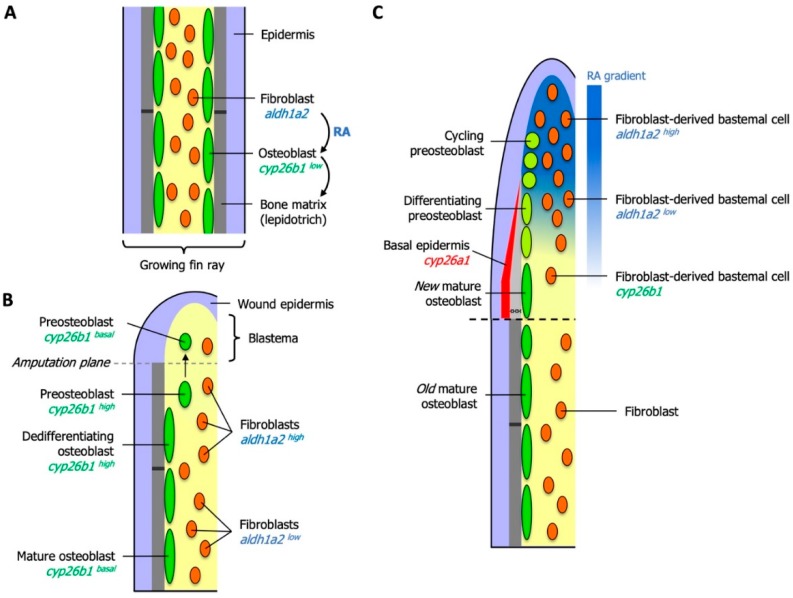
RA orchestrates bone growth during fin development and osteoblast behavior in regenerating fins. (**A**) As the fins grow, RA is produced by fibroblasts and stimulates matrix deposition (dark grey, black interruptions represent segmental joints) from osteoblasts in growing fin rays of juvenile and adult fish. Osteoblasts control exposure to RA by expressing *cyp26b1* at low enough concentrations to allow activation of bone matrix genes. (**B**) Immediately upon amputation, fibroblasts in undamaged stump tissue upregulate *aldh1a2* expression and flood the distal wound with RA. Osteoblasts need to protect themselves from RA by expressing *cyp26b1* in order to dedifferentiate to preosteoblasts and migrate into the blastema. (**C**) Regenerating fin rays set up an RA gradient that emanates from *aldh1a2* expressing distal blastema fibroblasts and fades out proximally by *cyp26b1* expressing proximal fibroblasts that act as a sink. Preosteoblasts divide in areas of high RA concentration and redifferentiate in areas below a certain RA threshold level. *cyp26a1* expression in cells of the proximal basal epithelial layer provides an RA-free niche that attracts preosteoblasts and allows end-to-end alignment of newly added osteoblasts with existing ones.

The zebrafish caudal fin is a well-studied model for understanding the cellular and molecular processes underlying fin growth and regeneration [166,167]. RA plays a general role in the normal growth of lepidotrichia (segmented rays of dermal bone): As bones grow in post-embryonic fins, RA is produced in fibroblasts and fosters the synthesis of bone matrix constituents from neighboring osteoblasts. Excessive signaling by RA, as in experimental situations, is counteracted by expression of *cyp26b1* in osteoblasts. Thus synthesis and degradation of RA in growing fins are tightly regulated [168] (Figure 3A).

Upon amputation, a proliferative blastema forms that consists of undifferentiated and proliferating cells that re-establish the fin and its skeleton. Precise control of the metabolism of RA and hence, the activity of RA signaling fulfills several important functions during regeneration. One of the first consequences of amputation is the elevated synthesis of RA through upregulation of *aldh1a2* in the stump fibroblasts. RA is required and sufficient to boost proliferation of stump cells and induce expression of the target genes *wnt10b* and *igf2b* in an autocrine fashion, whereas full activation of *fgf20a* expression also relies on other signals. Together, these genes promote the formation of the blastema [169].

Several signaling pathways have been shown to be required to ensure robust proliferation of cells in the blastema. When RA signaling is experimentally inhibited, both blastemal and epithelial cells show reduced proliferation rates. This is likely to be due to the breakdown of a network of RA-, FGF- and Wnt/β-catenin mediated signals that mutually stimulate each other’s activities. RA also down-regulates the growth-inhibitory effects of non-canonical *Wnt* signaling and thus is an integral part of the machinery that keeps the blastema in a proliferative state. Lastly, and in contrast to FGF, Wnt/β-catenin and Activin βA pathways, massive cell death is observed in cells of the blastema when the availability of RA is reduced [169], indicating that RA prevents cell death in a rapidly dividing tissue type. Regeneration of the zebrafish skeleton involves a substantial contribution from differentiated osteoblasts. In contrast, bone repair in mammals relies predominantly on mesenchymal stem cells [170]. Nonetheless, a thorough understanding of the dedifferentiation process in zebrafish osteoblasts informs efforts to improve bone healing in mammalian bone tissue.

### 6.2. Local Degradation of RA Controls Morphogenetic Processes of Osteoblasts and Osteoclasts

Osteoblasts in the regenerating fin are replenished from existing osteoblasts in the stump area and from a reserve population of osteoblast precursor cells [140,171]. Bone-forming osteoblasts are required to dedifferentiate before they become proliferative and migrate into the blastema [172,173,174]. During this process, differentiation markers are down-regulated and markers of immature osteoblasts are up-regulated. However, because high RA levels inhibit the dedifferentiation of osteoblasts to a proliferative preosteoblast state, osteoblast protect themselves from the effects of high local RA concentrations by rapid upregulation of the RA-degrading enzyme Cyp26b1, and this upregulation is not dependent on RA [168]. The inhibition of RA signaling in osteoblasts is thus one of the first mechanisms to be identified that regulates dedifferentiation during regeneration. Once preosteoblasts have migrated into the blastema, *cyp26b1* expression is shut down (Figure 3B). *aldh1a2* expression in the distal tip of the blastema provides a rich source of RA that supports blastemal proliferation and inhibits the redifferentiation of preosteoblasts.

As the proliferating blastema is displaced distally, fibroblasts in the proximal blastema express *cyp26b1*, thus acting as a sink that sharpens a distal-to-proximal RA gradient. The concept that Cyp26 enzymes can have cell non-autonomous consequences on RA levels within tissues has most clearly been demonstrated in experimental situations where cells reporting RA signaling lost the reporter signal when being transplanted into an environment of high Cyp26 activity, but not when surrounded by cells with low Cyp26 activity [27]. The principle has physiological importance, for example, during the formation of straight boundaries between rhombomeres (transiently forming segments) in the zebrafish hindbrain: When cells from rhombomeres (r) r3 or r5 intermingle with cells from an adjacent rhombomere during initial boundary development, higher Cyp26 expression in even-numbered rhombomeres subdues RA signaling in the stray cells and switches their identity to the appropriate fate [175]. Eventually, preosteoblasts align with osteoblasts in the most proximal blastema and redifferentiate into osteoblasts that extend the existing bone distally. These processes are triggered by an increase in distance between the RA source in the distal blastema and proximal preosteoblasts. In this environment, the concentration of RA falls below a threshold that allows osteoblast redifferentiation [168]. This elegant mechanism ensures a gradient of cells experiencing high and low levels of RA that allow the processes of proliferation (for the production of all cells that replace the lost structure) and redifferentiation of osteoblasts to run in parallel (Figure 3C).

Re-formed osteoblasts have to accurately align with existing skeletal structures. To achieve this, preosteoblasts proliferate locally under the influence of a proximally restricted source of *Shha* that originates in the epidermis [176]. In order for *shha* to be transcribed, proximal parts of the basal epidermal layer have to be cleared from RA, which is achieved through the expression of another Cyp26 gene, *cyp26a1*(Figure 3C). An experimentally induced loss of RA clearance results in seemingly random migration of osteoblasts into interray or even stump tissue. Osteoblasts may themselves exert a piloting function for other cell types, as the breakdown of ray–interray boundaries also affects other cell types, like fibroblasts and blood vessels [176]. An excess of RA results in a similar phenotype and induces an over-mineralized phenotype, by promoting bone matrix synthesis in osteoblasts [177]. Suppression of RA signaling by removing RA locally, as observed in the stump and in the proximal blastema, is a mechanism repeatedly utilized to guide osteoblast behavior towards the correct regenerative morphogenetic processes. Experimentally elevated RA levels during osteoblast differentiation in regenerating fins also results in irregularly shaped hemirays [168]. This finding led to the observation that regeneration of new bone is accompanied by osteoclasts accumulating at the inner and outer surfaces of newly forming bone matrix. Although RA is known to inhibit the differentiation of osteoclasts [40,178,179], RA levels are low enough in the proximal blastema for osteoclasts to remove excess matrix to define the final shape of new hemirays.

### 6.3. RA Controls Cell Fate in the Preosteoblast Lineage

Another interesting role for RA has been identified in controlling cell fate choice in the preosteoblast lineage [180]. The fin ray skeleton is formed by osteoblasts and is subdivided by bone articulations, or joints, at regular intervals. Joints are formed during growth, and reformed during regeneration, by a distinct cell type—the joint-forming osteoblasts. These are aligned in two rows, one each on either side of a new articulation [181]. Joint-forming osteoblasts and (regular) osteoblasts originate from a common preosteoblast cell lineage [180]. Preosteoblasts that express *runx2a/b* differentiate into osteoblasts, while those expressing *evx1*, *hoxa13* and *pthlha* become committed to forming joint cells. RA treatment during regeneration suppresses joint cell markers. The effect might act directly on joint cells, since they express the RA-receptor *rargb* and because mature joint-forming osteoblasts down-regulate expression of their lineage markers under RA-treatment. Prolonged RA-exposure of mature fin rays also leads to the appearance of new osteoblasts in the joints. Reporter gene analyses showed that the fate of mature joint-forming osteoblast is not fixed, instead they differentiate to (regular) osteoblasts under RA, presumably by lifting an arrest in osteoblast differentiation or by transdifferentiation. If this effect contributes significantly to the over-ossification observed in RA-treated regenerates [176,177] has not been established yet. The findings underscore once more the requirement for precise spatio-temporal control of RA signaling during fin growth and regeneration.

### 6.4. Growth Control Upstream of RA in Zebrafish Fins

Proximal parts of the caudal fin regenerate faster and with a proportionately larger blastema than more distally located parts, a mechanism that allows the regenerative growth of proximally injured parts of the fin to catch up with the distal edge. This phenomenon is known as allometric growth and contrasts with isometric growth, which preserves proportional relationships in a growing organism. Fin growth rates are controlled by the protein phosphatase Calcineurin. When the Calcineurin inhibitor FK506 is applied to the regenerating fin, it switches to allometric growth mode, typical for proximal regeneration. Thus, the role of active Calcineurin signaling is to enable a slower, isometric growth rate [182]. Calcineurin exerts its effect on regeneration rates by negatively controlling RA signaling. When Calcineurin is inhibited, *aldh1a2* expression as well as *rarg* and *crabp2b*, which binds RA and increases RA availability to nuclear receptors [183], are up-regulated in the blastema, even prior to visible proximal allometric regeneration [182]. Conversely, genes involved in the degradation of RA signaling, *cyp26a1*, *cyp26c1* and *crabp2a*, which transports RA to Cyp26 enzymes for degradation [28], are down-regulated in the blastema when Calcineurin is inhibited. Increased RA signaling has been shown to increase proliferation rates in the blastema [169]. Calcineurin directly regulates members of the NFAT transcription factor family [184], but another target has been identified in fins that control RA-mediated growth. The *another long fin (alf)* mutant develops with overgrown fins that have elongated skeletal segments, a phenotype that is indistinguishable from FK506-treated fins. *alf* encodes the two-pore domain potassium (K^+^) channel Kcnk5b [185] and is thought to be a gain-of-function mutant, because loss-of-function mutants in *kcnk5b* possess and regenerate normal fins without overgrowth. Together, this suggests that Calcineurin might act to inactivate Kcnk5b. Calcineurin is thought to bind to the Kcnk5b C-terminus that, when mutated, results in Kcnk5b losing sensitivity to Calcineurin. Mutants that lead to the loss of the last transmembrane domain, which also harbors the point mutation in *alf*, and the C-terminal end result in overgrowth phenotypes. How changes in the membrane potential of fin tissue affect RA signaling activity remains to be examined.

Does RA control position in the zebrafish fin or does it control growth rates? Because of the proximalizing activity that RA exerts on regenerating limbs in salamanders [186,187] and the fact that fin ray bifurcations (as presumed markers of proximo-distal identity) are shifted distally in RA-treated regenerates [188,189], RA was believed to control proximal identity in the fin. However, FK506 treatment, which leads to upregulation of RA signaling, does not proximalize fins, because removal of the drug results in an immediate stop of regenerative growth rather than continuation of an allometric growth program (which would be expected if the fin was proximalized) [190]. Instead, accelerated growth requires the continued presence of the drug. Also, fins that regenerated under the influence of FK506 to a larger fin do not regenerate to the enlarged size when resectioned without the presence of FK506. It is most likely, therefore, that RA signaling activity controls growth rates rather than positional values.

## 7. Conclusions

Research into RA signaling in development remains a highly productive field that experiences continuous advances and has led to an enhanced understanding of the underlying mechanisms and principles. One principle that continues to resurface in various contexts is the formation of morphogen gradients of RA that determine cell fate decisions in a concentration-dependent manner. Prominent examples that are well characterized include the hindbrain, placode- and neural crest-derived craniofacial structures as well as the paraxial mesoderm and neural tube [5,21,191,192]. During vertebrate trunk development, newly generated mesodermal cells synthesize RA, which, in a gradient opposing that of Wnt signals, determines the rate at which NMPs are produced and induces neural differentiation. *Cyp26a1* is expressed dynamically in the caudal-most region that includes the NMPs and keeps RA at a low concentration, which is an absolute requirement for the differentiation of NMPs to the mesodermal lineage. RA signaling thus coordinates the production of neural and mesodermal tissue. Local sources and sinks of RA have also been identified in the regenerating zebrafish fin, where fibroblasts of the proliferating blastema in the distal regenerate provide a source of RA, while fibroblasts in the proximal regenerate express *cyp26b1*. Preosteoblasts in the emergent RA gradient proliferate distally in a “high RA” environment and redifferentiate proximally to osteoblasts in a “low RA” environment. As in the vertebrate trunk, the RA gradient is highly dynamic, in that it advances distally and leaves new osteoblasts in its wake that rebuild the fin ray skeleton. Local gradients of RA may also underlie the extent of tooth initiation in the pharyngeal region of zebrafish, since RA treatments initiate the formation of ectopic teeth in anterior and dorsal pharyngeal positions. However, the responsible sources and sinks remain to be characterized in more detail.

Another important principle is that Cyp26 activity in one cell type can act as a local sink to keep RA below a threshold concentration in a neighboring cell type. We reviewed examples during vertebrae development in zebrafish, where RA induces a reiterative pattern of axial *entpd5a*/*cyp26b1* expression in chordoblasts, which eventually causes a segmented mineralization of the notochord sheath and formation of chordacentra. Here, expression of *cyp26b1* acts as a sink for RA that apparently keeps neighboring areas, the future intervertebral discs, RA-free and thus prevents mineralization. It should be noted, however, that the exact sources for RA remain to be resolved in future studies. The RA gradient in the regenerating zebrafish fin serves as another example for non-autonomous loss of RA signaling, because *cyp26b1* expressing fibroblasts eliminate RA from their environment to allow neighboring preosteoblasts to drop out of the cell-cycle and differentiate again. Finally, fin regeneration also presented examples where cells use cell-autonomous inhibition of RA signaling to protect themselves from unwanted effects in a “high RA” environment: Basal epidermal cells eliminate residual RA to ensure appropriate signaling activities that attract osteoblasts by expressing *cyp26a1* and stump osteoblasts express *cyp26b1* to undergo dedifferentiation in an otherwise proliferation-enhancing environment rich in RA, where both processes are mutually exclusive for osteoblasts. Ongoing efforts from mammalian and non-mammalian vertebrate model systems are expected to shed light on RA signaling from a developmental and evolutionary point of view. The work in zebrafish, whose developmental mechanisms do not always closely match those in tetrapods, sheds light on the scope of evolutionary modifications that changes in RA-mediated gene regulation has facilitated. It is also informative with regard to identifying developmental processes that may have been overlooked in mammalian model systems.
